# Correction: Ploidy and recombination proficiency shape the evolutionary adaptation to constitutive DNA replication stress

**DOI:** 10.1371/journal.pgen.1010864

**Published:** 2023-07-28

**Authors:** Marco Fumasoni, Andrew W. Murray

The Funding statement contains an error. The correct Funding statement is:

MF was supported by fellowships from The European Horizon 2020 Framework Programme, H2020-MSCA-IF-2020–101030203/GENMAINEVO (https://ec.europa.eu/), the Human Frontier Science Program—LT000786/2016-L (https://www.hfsp.org/), the European Molecular Biology Organization—ALTF 485–2015 (https://www.embo.org/) and the Associazione Italiana per la Ricerca sul Cancro—iCARE 17957 (https://www.airc.it/). AWM was supported by grants from the National Institute of General Medical Sciences—GM43987/RO1 (https://www.nigms.nih.gov/), the National Science Foundation—#1764269 (https://www.nsf.gov/) and the Simons Foundation—#594596 (https://www.simonsfoundation.org/). The funders had no role in study design, data collection and analysis, decision to publish, or preparation of the manuscript.
