# Supplementary figures and images for: Ploidy and recombination proficiency shape the evolutionary adaptation to constitutive DNA replication stress
Source: PLoS Genet. 2021 Nov 9;17(11):e1009875. doi: 10.1371/journal.pgen.1009875 (PMC8604288; doi:10.1371/journal.pgen.1009875)

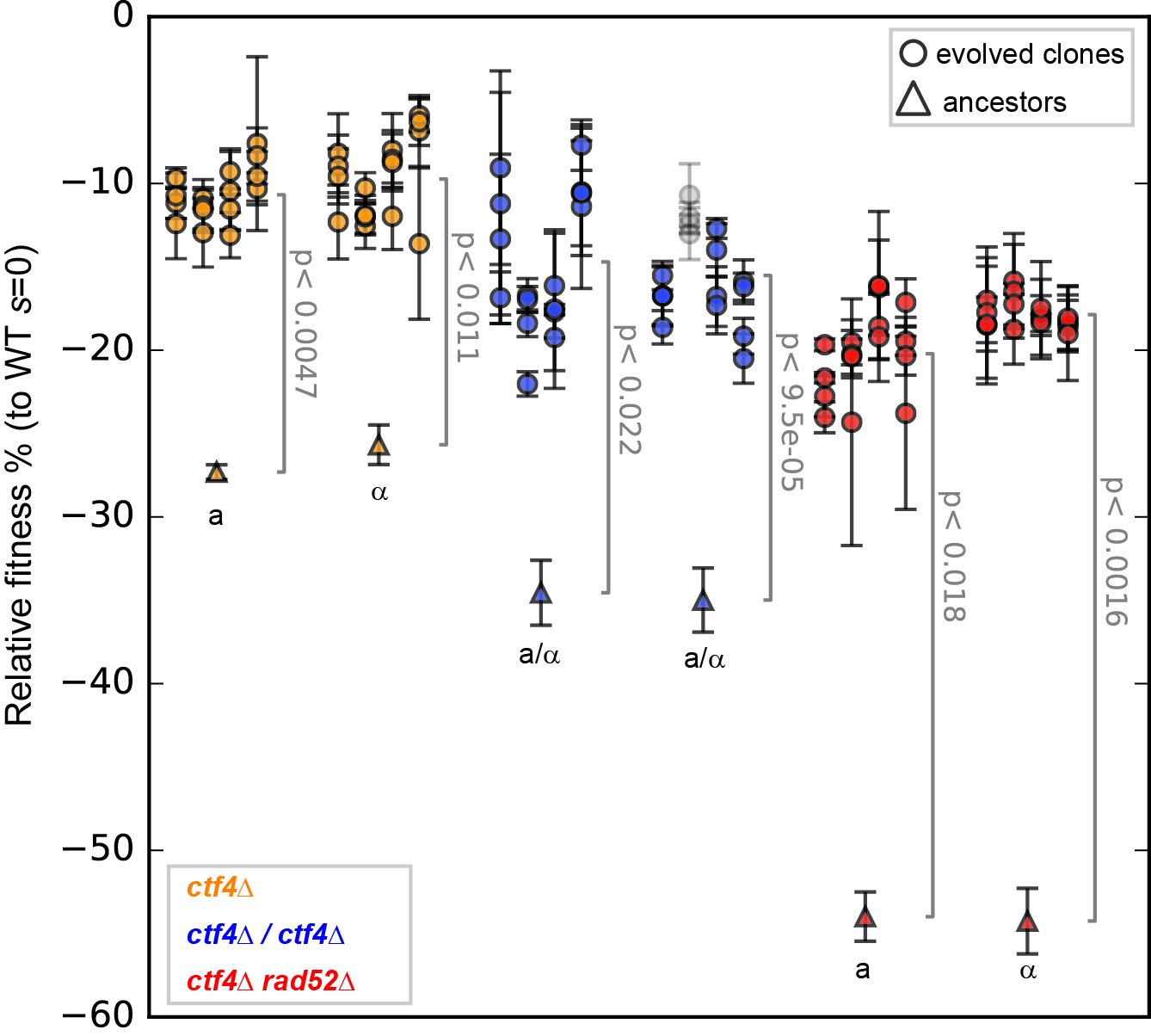

Supplement: S1 Fig — Fitness of 96 clones isolated from the 24 evolved populations (4 clones for population, distributed on the same vertical line), relative to haploid or diploid WT cells (s = 0). Fitness data of haploid strains (orange) is from [17]. Semi-transparent grey dots represent clones that became haploid over the course of the experiment. Error bars represent standard deviations. a and α refer to the strains’ mating type (MAT locus). a/α indicates diploid strains. The P-values reported in figures are the result of t-tests assuming unequal variances (Welch’s test). The fitness values shown here are reported in S2 Data. (TIF) [file pgen.1009875.s001.tif]

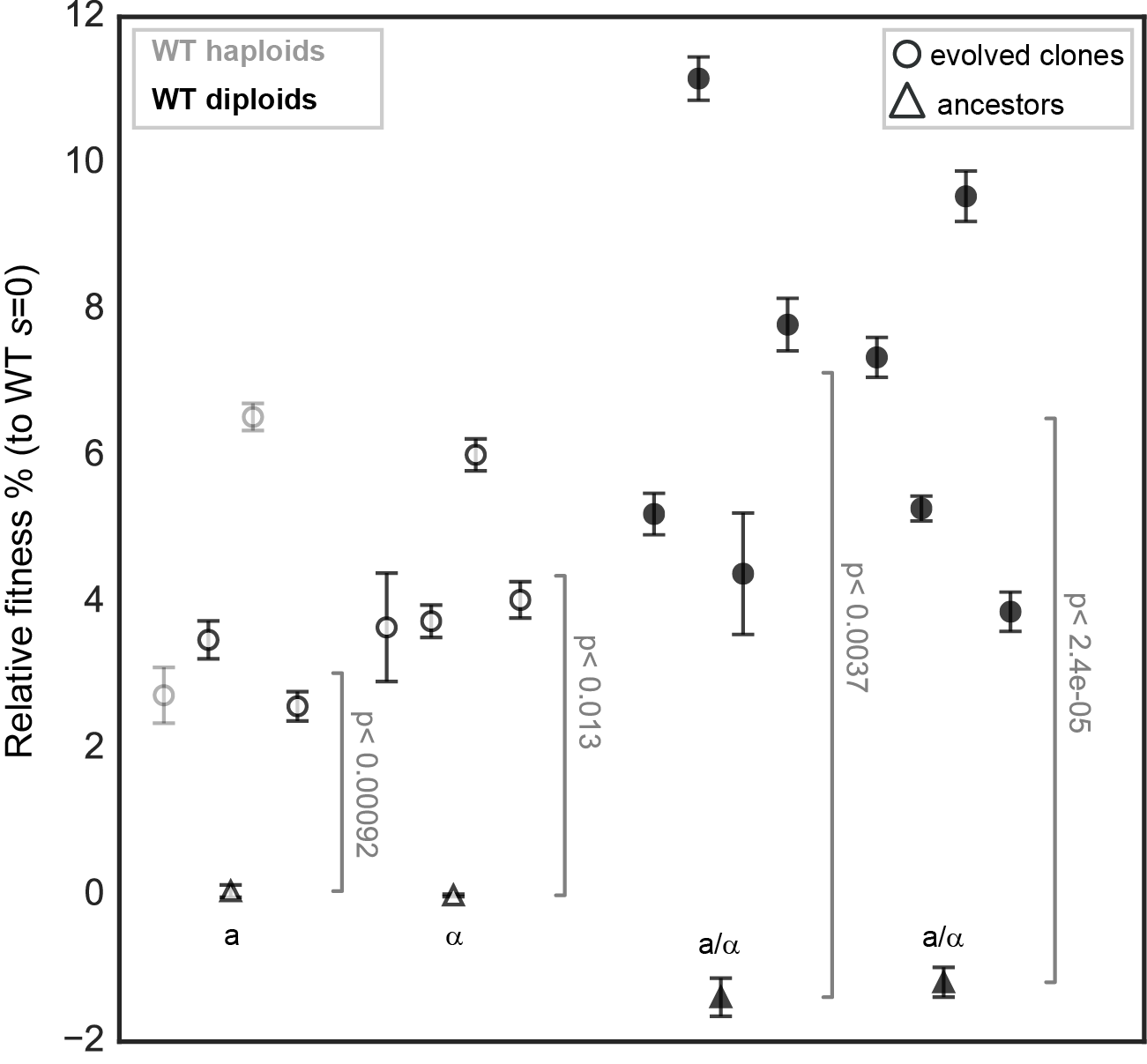

Supplement: S2 Fig — Fitness of the WT haploids (white) and diploids (black) ancestors and of 16 evolved populations derived from them (four from each ancestor), relative to WT cells (s = 0). Fitness data of haploid strains (white) is from [17]. Semi-transparent gray dots represent populations that changed ploidy over the course of the experiment. Error bars represent standard deviations. a and α refer to the strains’ mating type (MAT locus). a/α indicates diploid strains. The P-values reported in figures are the result of t-tests assuming unequal variances (Welch’s test). The fitness values shown here are reported in S1 Data. (TIF) [file pgen.1009875.s002.tif]

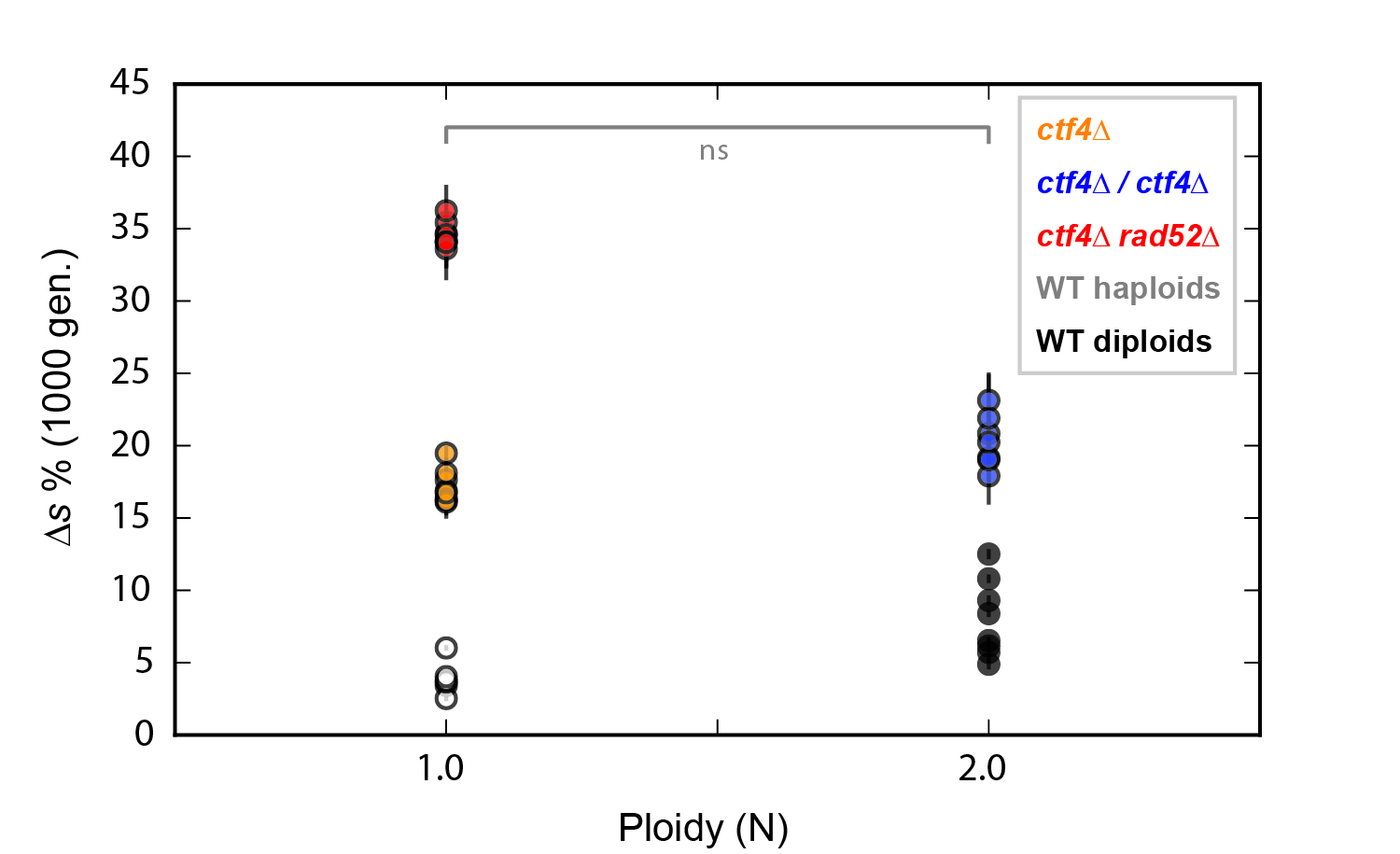

Supplement: S3 Fig — Fitness increase over 1000 generations (Δs, measured as the difference between the populations’ final fitness and the fitness of their respective ancestors that lacked Ctf4) relative to the ploidy of the ancestor cells (N). Fitness data of haploid strains (orange and white) is from [17]. Error bars represent standard deviations. The P-values reported in figures are the result of t-tests assuming unequal variances (Welch’s test). The data shown here are reported or derived from S1 Data. (TIF) [file pgen.1009875.s003.tif]

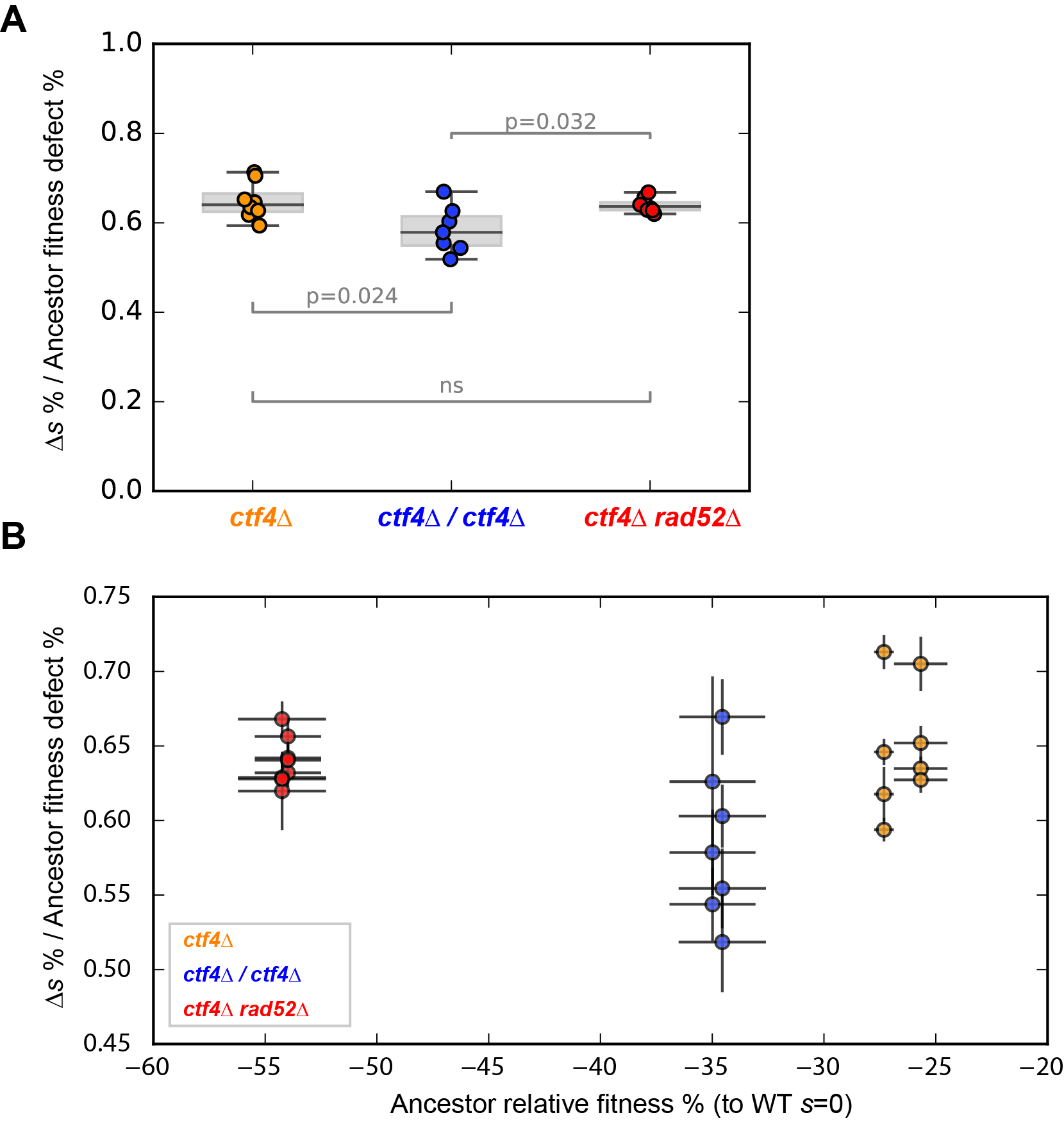

Supplement: S4 Fig — (A) The populations’ fitness increase over 1000 generations (Δs) was divided by their ancestors’ fitness defect (relative to strains of the same ploidy containing Ctf4) to calculate the fraction of the initial defect that was recovered during the experiment. (B) Fraction of the initial fitness defect recovered over the course of the experiment, relative to the ancestor’s fitness. Fitness data of haploid strains (orange) is from [17]. Error bars represent standard deviations. The P-values reported in figures are the result of t-tests assuming unequal variances (Welch’s test). The data shown here are reported in or derived from S1 Data. (TIF) [file pgen.1009875.s004.tif]

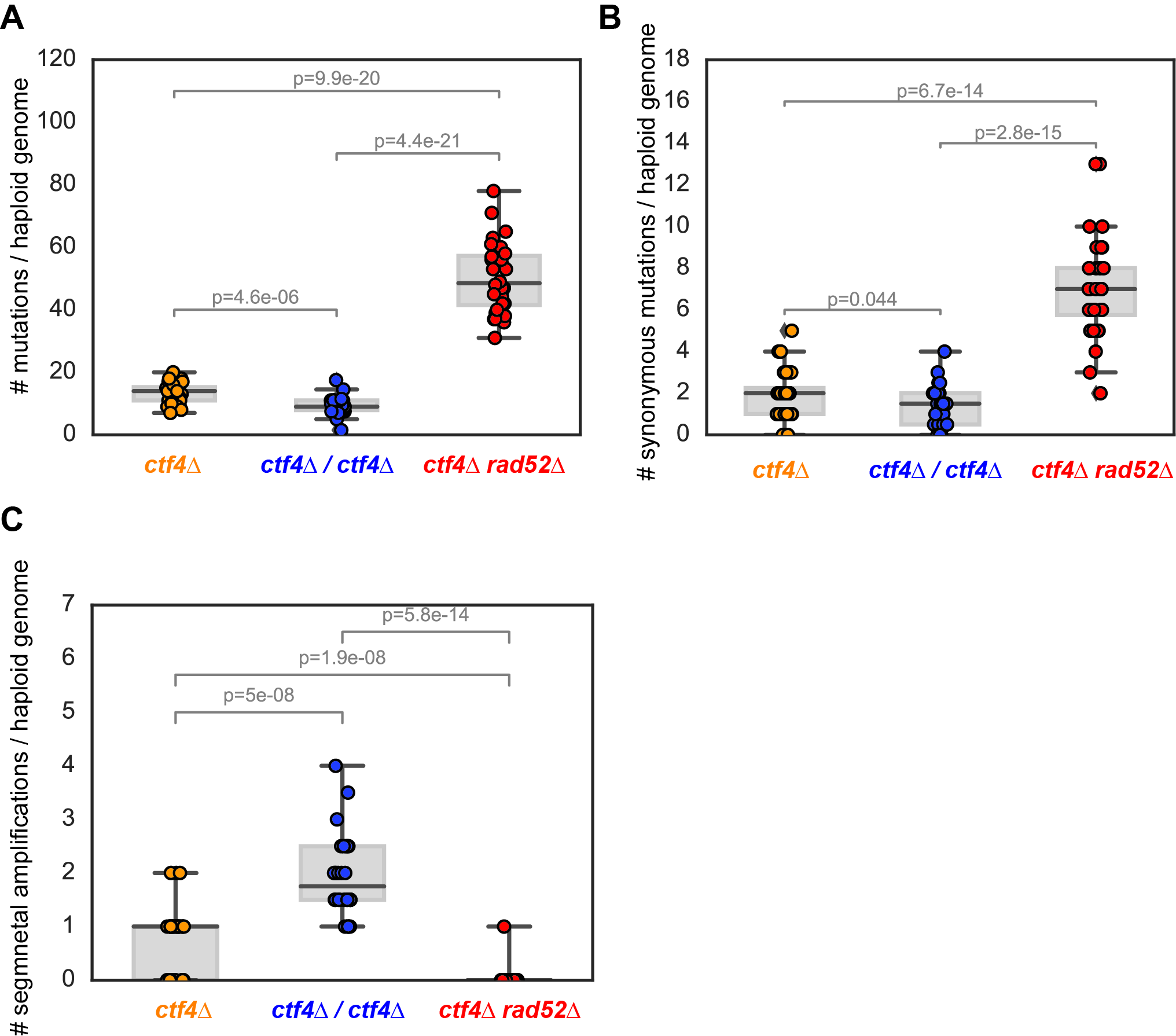

Supplement: S5 Fig — (A) Total number of mutations per haploid genome detected in the clones of each of the three strains with different genomic features. (B) Total number of synonymous mutations per haploid genome for each strain. (C) Total number of segmental amplifications per haploid genome for each strain. The P-values reported in figures are the result of t-tests assuming unequal variances (Welch’s test). The values shown in A and B are reported in S4 Data. Values shown in C are derived from S4 Table. (TIF) [file pgen.1009875.s005.tif]

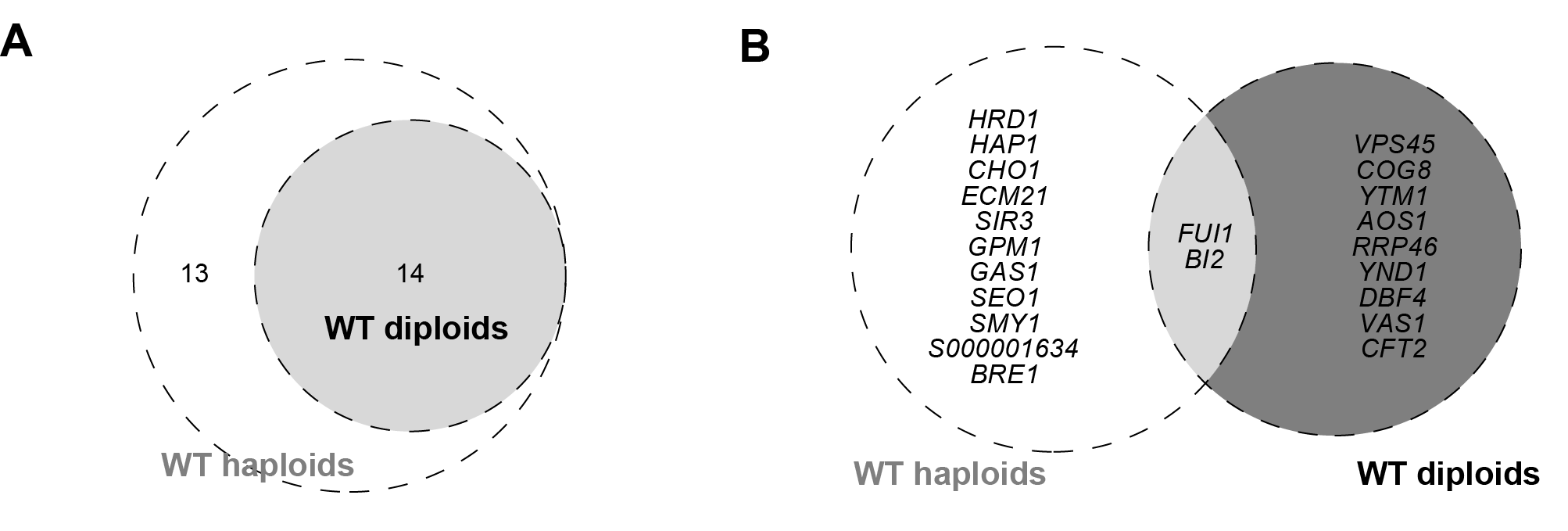

Supplement: S6 Fig — (A) Venn diagram representing the mutations found in the wild-type evolved populations. The circles’ areas are proportional to the unique list of genes found mutated in the haploid or diploid evolved WT populations. 14 genes were mutated in both diploids and haploids and 13 were mutated only in haploids. (B) Venn diagram of the genes in which mutations were significantly selected. Notes that all the 14 mutations detected in the evolved diploids were also present in the evolving haploids populations (panel A), but the 9 genes listed in most right sector in panel B were only significantly selected in evolved diploids. (TIF) [file pgen.1009875.s006.tif]

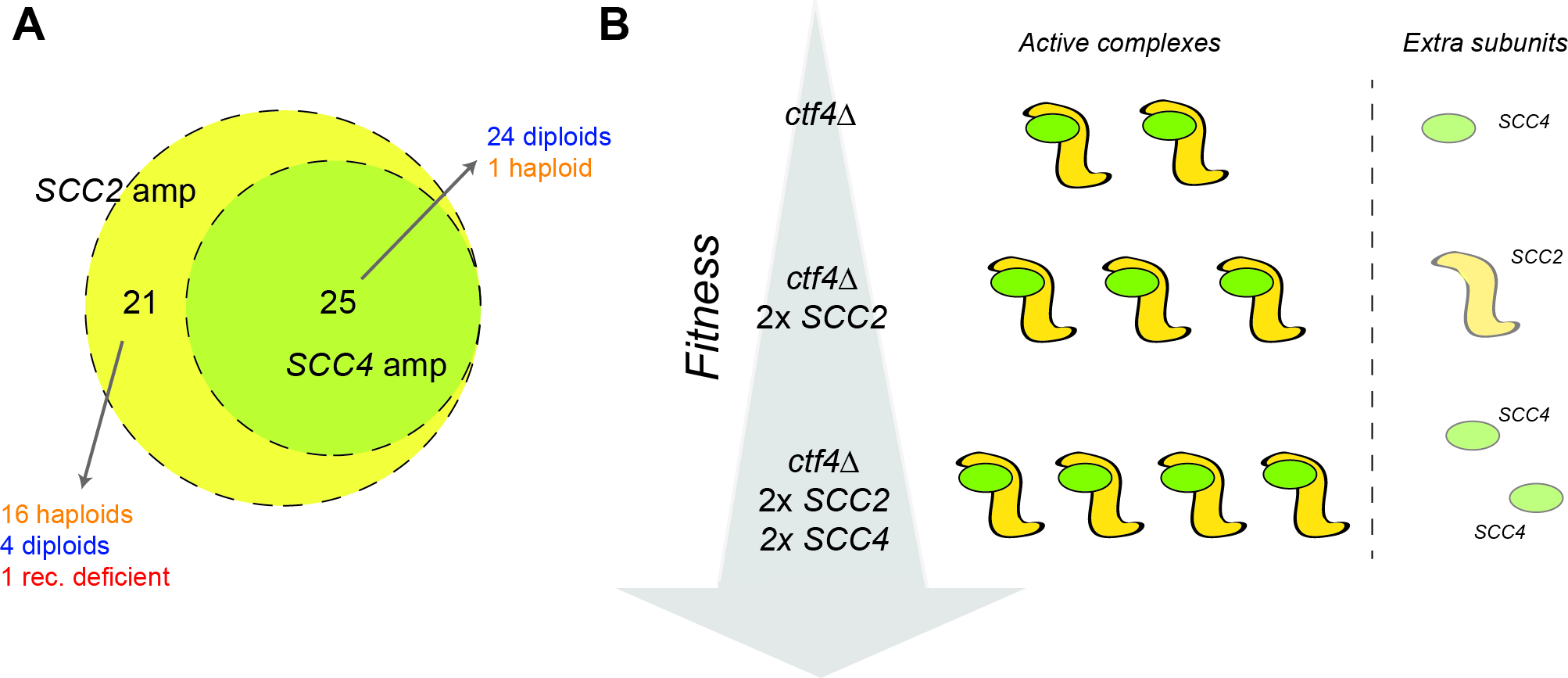

Supplement: S7 Fig — (A) Venn diagram representing the number of clones carrying SCC2 (yellow) and SCC4 (green) amplifications. Note how all clones carrying an SCC4 amplification also have a SCC2 amplification, and that out of the 25 clones that had amplified both genes, 24 were detected in diploid populations. (B) Cartoon of our model for the amplification of cohesion loaders during adaptation to constitutive DNA replication stress: Scc2 is initially the limiting subunit for the formation of active complexes. The initial amplification of SCC2 produces extra Scc2 protein and thus increases the number of active cohesin loaders and makes Scc4 the limiting component. The subsequent amplification of SCC4 provides extra binding partners and further increases the number of active complexes available. (TIF) [file pgen.1009875.s007.tif]

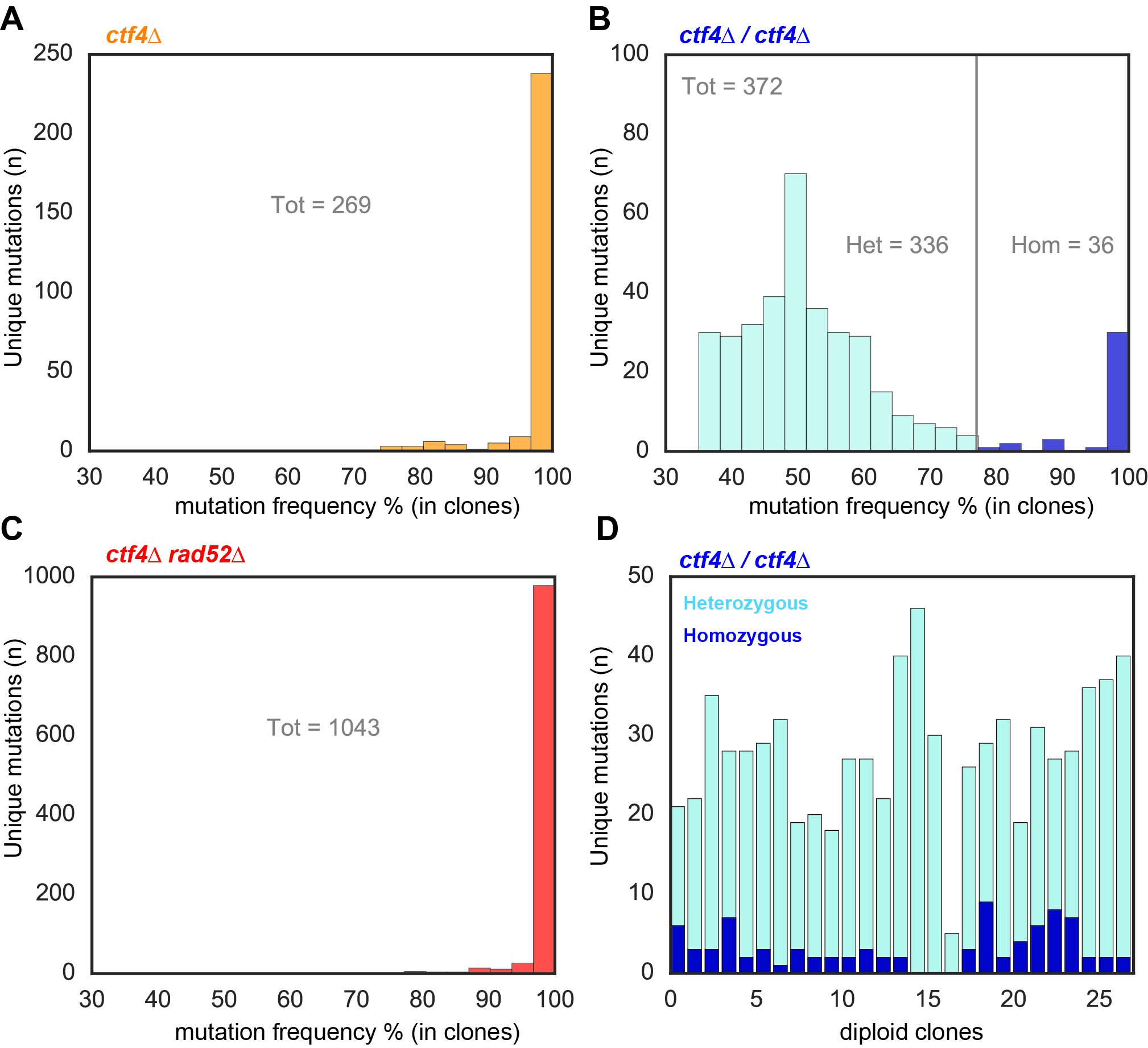

Supplement: S8 Fig — Frequencies of mutations in (A) ctf4Δ (B) ctf4Δ/ctf4Δ and (C) ctf4Δ rad52Δ evolved clones were obtained from the ratios of mutated to total DNA reads covering the locus. Mutations present in more than 77% of the reads were considered homozygous (Hom) in ctf4Δ/ctf4Δ diploids. Mutations present in less than 77% of the reads were considered heterozygous (Het). (D) Number of heterozygous (light blue) and homozygous mutations (dark blue) found in each individual, sequenced, evolved ctf4Δ/ctf4Δ diploid clone. All the values shown here are derived from S1 Table. (TIF) [file pgen.1009875.s008.tif]

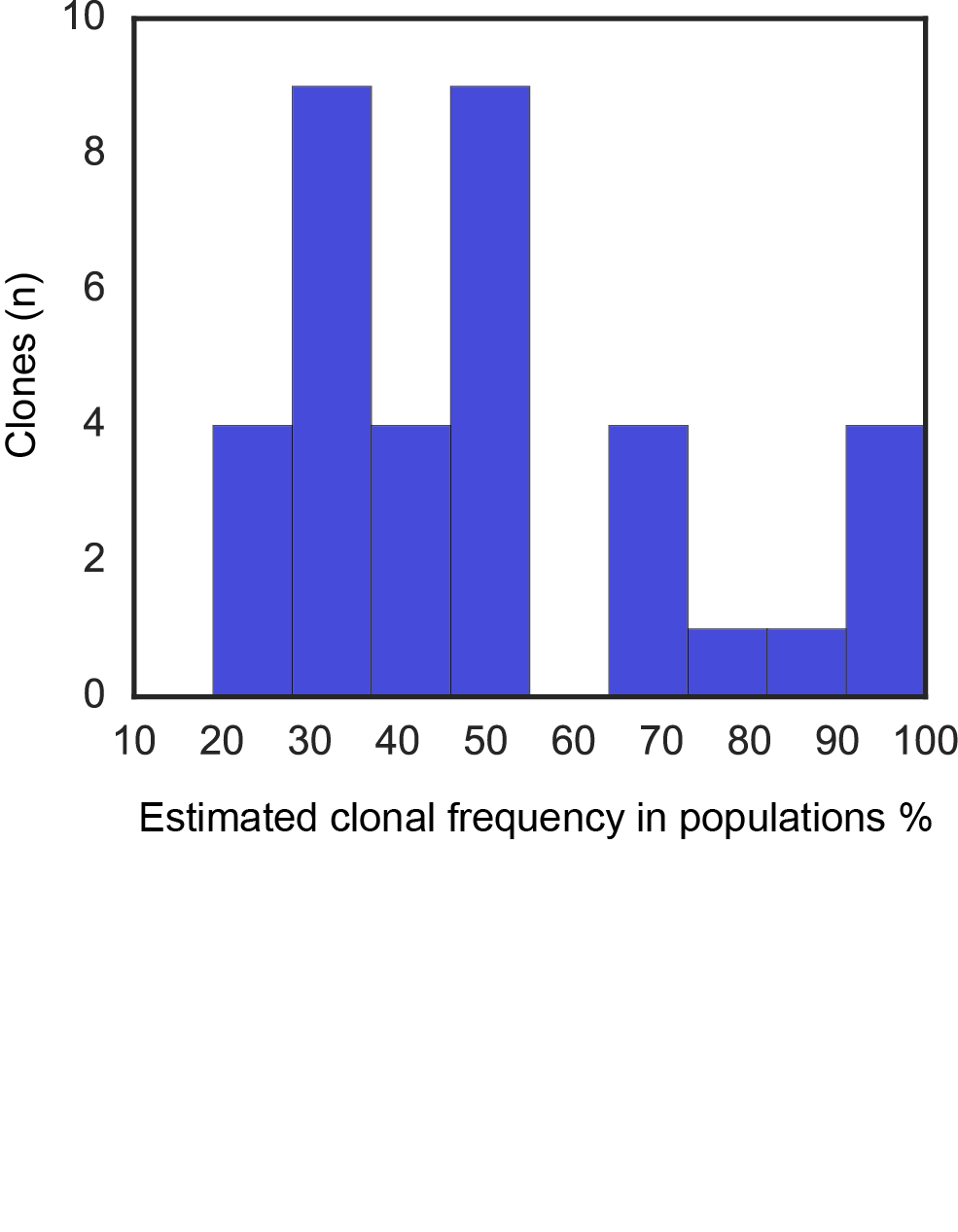

Supplement: S9 Fig — Clonal frequency estimates within populations were derived from the percentage of reads from whole population sequencing that carried those mutations previously identified, in clones, as homozygous. All the values shown here are derived from S1 Table. (TIF) [file pgen.1009875.s009.tif]

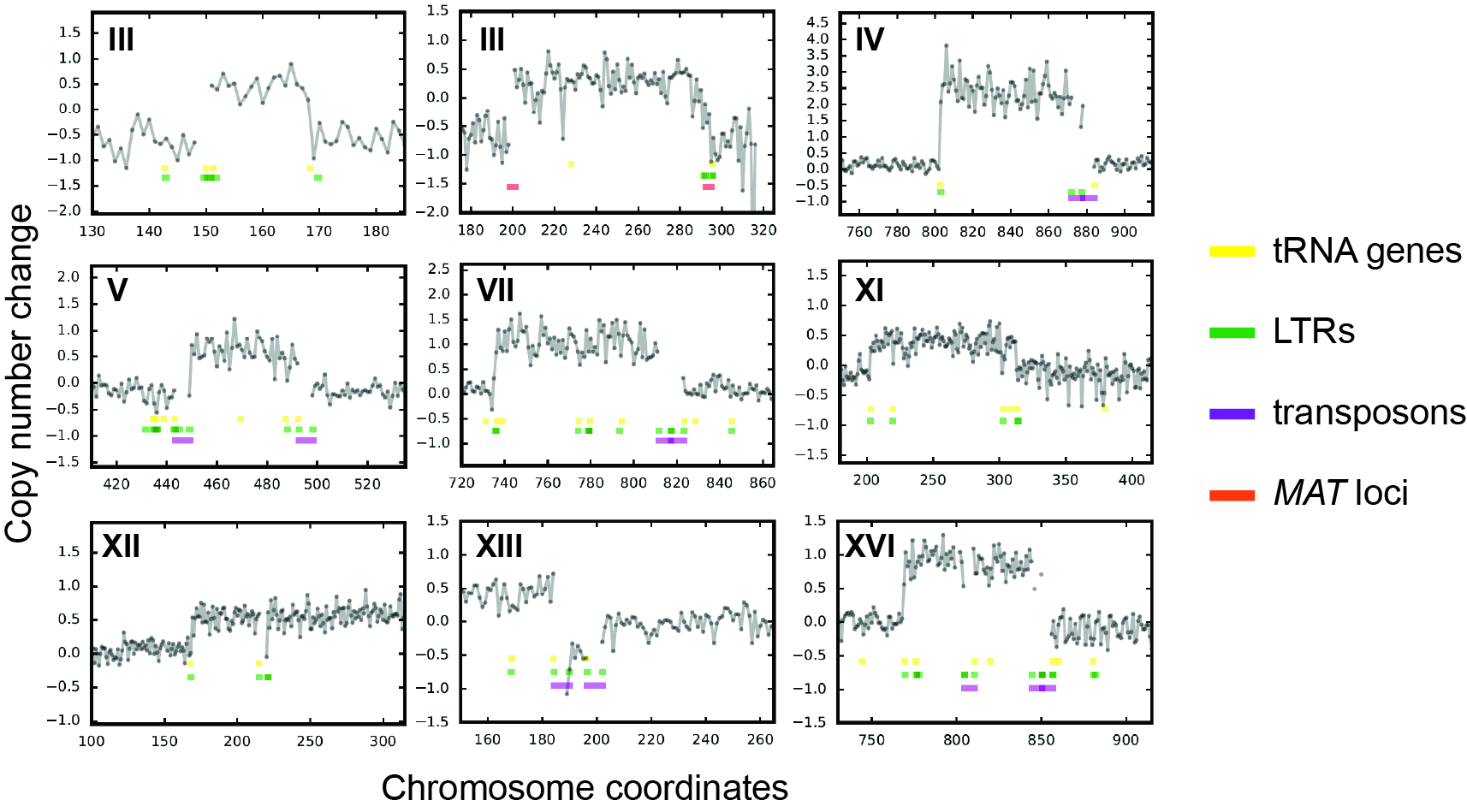

Supplement: S10 Fig — Magnification on the most recurrent copy number variations (CNVs) affecting different chromosomes (roman numbers). Repetitive sequences present in the surrounding chromosomal coordinates are noted in different colors: tRNA genes in yellow, Long Terminal Repeats (LTRs) in green, transposons in purple and MAT loci in red. Copy number change refers to the fragment’s gain or loss during the evolution experiment (i.e. +1 means that one copy was gained in haploid cells, and that two copies were gained in diploid cells). (TIF) [file pgen.1009875.s010.tif]

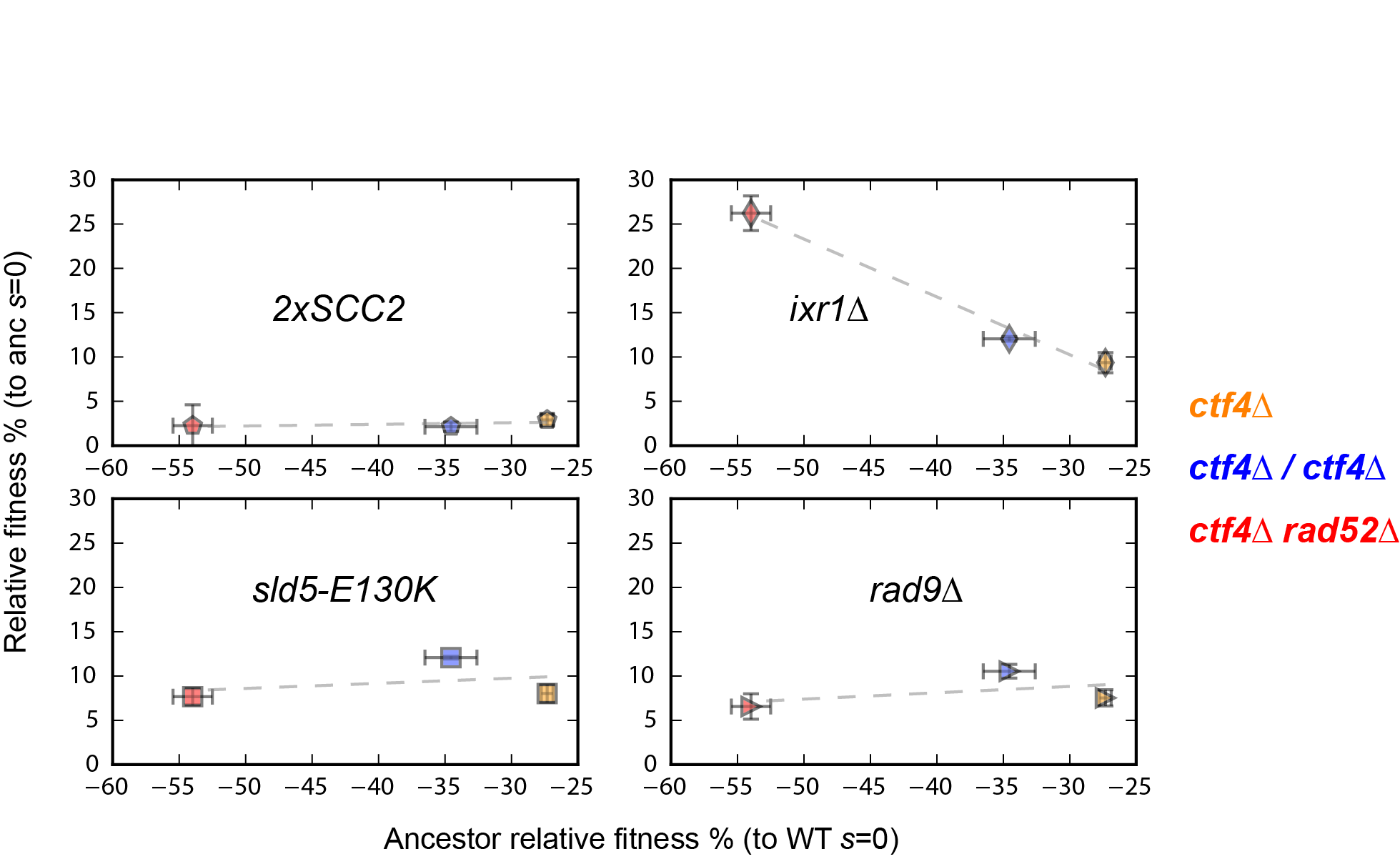

Supplement: S11 Fig — The fitness increase provided by four adaptive mutations reconstructed in strains with different genomic features versus their genetic background’s ancestral fitness defect. Fitness data of haploid strains (orange) is from [17]. Error bars represent standard deviations. The fitness values shown here are reported in S6 Data. (TIF) [file pgen.1009875.s011.tif]

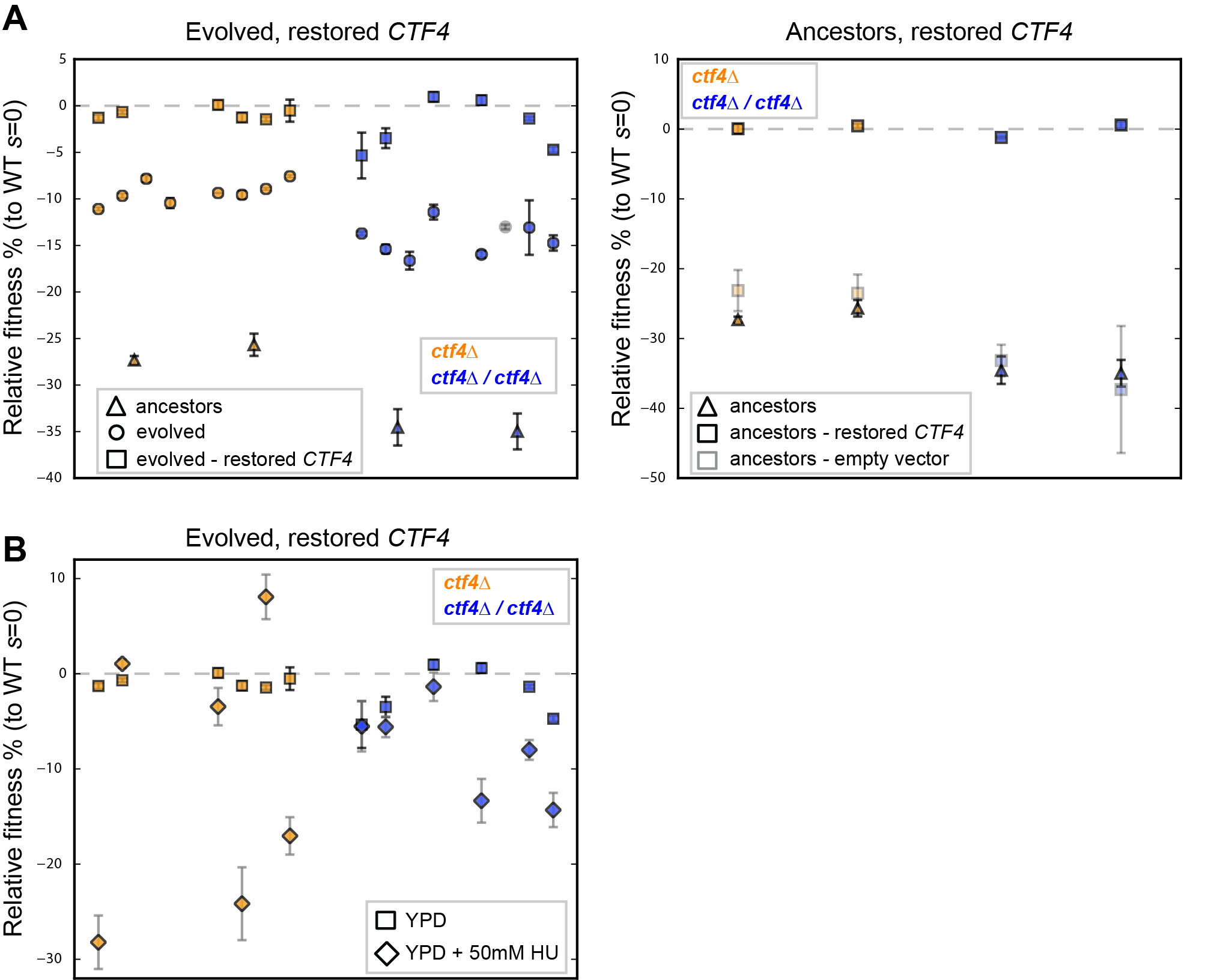

Supplement: S12 Fig — A wild type CTF4 gene was reintegrated into the genome of evolved cells. The integration was not possible in populations 2,3 and 11, which became prototrophic for the marker used for the integration (URA3) and in all recombination-deficient strains, because of their inability to recombine an exogenous DNA fragment into their genome. (A) Fitness of the CTF4-restored evolved populations in YPD (left panel). Fitness of the CTF4-restored and empty-vector transformed ancestors (right panel). (B) Fitness of the CTF4-recovered evolved populations in the presence and absence of 50mM Hydroxyurea. The points in the absence of hydroxyurea also appear in A. The fitness values shown here are reported in S9 Data. (TIF) [file pgen.1009875.s012.tif]
