# Supplementary material for: Ploidy and recombination proficiency shape the evolutionary adaptation to constitutive DNA replication stress
Source: PLoS Genet. 2021 Nov 9;17(11):e1009875. doi: 10.1371/journal.pgen.1009875 (PMC8604288; doi:10.1371/journal.pgen.1009875)
Supplement: Attachment — Submitted filename: PlosGenetics_Response.docx [file pgen.1009875.s033.docx]

Dear Editors and Reviewers,

Thank you for the careful and through reviews our article. We are particularly grateful to the PLOS Genetics editorial team, which granted us an extension to the revision period. This allowed the first author the time to set up a new lab in a different continent, where the experiments for the revision have been conducted. We deeply appreciate the understanding shown by PLOS genetics, especially at a time where many additional challenges in setting up a new lab have to be faced as a consequence of the persisting pandemic.

We were very pleased to hear that the reviewer #1 considered our work solid and suggests publication. Reviewer #2 questioned the novelty of our work, and suggested a number of additional experiments that would improve our paper.

We believe our work is novel in two important ways, which we emphasize in the revised manuscript. First, ours is the first analysis of the evolutionary response to the same genetic damage in three different genomic contexts: haploids, recombination-deficient haploids and diploids. Second, we have advanced the analysis of the genetic basis of adaptation in experimental evolution by reconstructing a complex evolutionary adaptation in a diploid organism, and testing the phenotype of individual adaptive mutations in all three genomic contexts, including backgrounds where these mutations were not observed. The major conclusions of the paper depend on this level of analysis.

We have also performed many of the experiments and analyses suggested by reviewer #2. These include analyzing the dynamics of adaptation of the various populations and studying the phenotype of mutations and combinations of mutations in the in the absence of the perturbation that selected for them (*ctf4Δ*).

We are grateful for the reviewers’ contributions to our work, which we believe have improved both the accuracy and the relevance of our study. The changes to the text are highlighted in accompanying Word document. Below we provide detailed responses to the points raised by the reviewers.

Sincerely,

Marco and Andrew

Reviewer's Responses to Questions

**Comments to the Authors:**

**Please note here if the review is uploaded as an attachment.**

**Reviewer #1:** This work is a follow-up of the authors’ previous work. Previously, the authors studied how haploid yeast cells adapted to DNA replication stress (*CTF4* deletion) using experimental evolution. In the current work, the authors did the same experiment in two other genetic backgrounds (diploid cells and recombination-deficient haploid cells). Overall, the adaptive mutations accumulated in the three strains share some similarity, this is further proven by Figure 6, where the fitness of the ancestors is fully restored by introducing the same set of mutations to all three strains. However, the authors also found some adaptive mechanisms that are specific to each genetic background. For example, the authors reported the “two-step mutations” (a mutation followed by a LOH event) in diploids and there is almost no gene amplification in recombination-deficient strain. In conclusion, I think this is a solid work that deserves a publication and I recommend a minor revision. My specific questions are listed below:

1. The authors define ploidy and rad52-deletion as “genome architecture”. I think they are just different genetic backgrounds, not different genome architectures in the strict sense. I agree with the definition of genome architecture I found online: “Genome architecture describes the spatial arrangement of the functional and regulatory elements of a genome.” I’m not an expert on terminology, but I think it would be better to use a more accurate term.

We recognize the potential confusion generated by the difference between the way we defined ‘genome architecture’ in this manuscript, and some of the definitions present in the literature. We thus decided to employ ‘genomic features’ as a term to refer, generically to variation in properties such as ploidy and recombination proficiency.

2. In lines 133-135, the authors mentioned that wildtype haploid and diploid are evolved in the same environment to control for mutations that confer environment-specific benefit. But this data is not used in the later analysis.

Thank you for pointing out this omission. We did explain how we use the data derived from wild type evolution in Material and methods (lines 730-737), but we agree that this information should be mentioned in the main text. We now include a supplementary figure depicting the mutations detected in evolved wild-type populations (Figure 3S2), and the following text in lines 214-217:

*“Genes whose mutations were significantly selected in the evolved wild-type populations were removed from the list of evolved ctf4Δ strains to control for mutations selected by the environment of the experiment, rather than the absence of Ctf4 (Figure 3S2, see material and methods for details).”*

3. Does *CTF4* deletion affect spontaneous mutation rate? Maybe compare the number of mutations accumulated in *CTF4*-deletion strain with the wildtype.

*Ctf4Δ* cells manifest a 5x fold increase in mutation rates compared to wild-type cells, as reported previously in Fumasoni et al., Mol. Cell., 2015. We now include this information in the main text (Line 482-483, see below)

4. The mutation rate of the rad52 deletion strain is much higher than the other two, this feature should also contribute to the faster evolution of the recombination-deficient strain. This point should be mentioned.

We agree with reviewer #1 and we now include a paragraph commenting on the effect of mutation rates on the adaptation rates (Line 481-489):

*“The different mutations rates in strains with different genomic features may affect the adaptation rates of the various populations. Cells lacking Ctf4 were previously reported to have a 5-fold increase in their mutations rate [49], and we found an additionally 4-fold increase in recombination-deficient strains (Figure 3S1B). Nevertheless, we believe these differences play a secondary role in determining the adaptation rates. First, despite having a higher mutations rate, the recombination-deficient strains adapt at a similar pace to haploid and diploid strains for much of the experiment (Generation 100 to 1000, Figure 2A, 2C). Second, slower adaptation in diploids is not limited to populations experiencing DNA replication stress (Figure 2A) but is also present in control wild-type populations (Figure 2C), which do not have altered mutations rates.”*

5. The citation of Figure 2C (line 171) in the MS appears early than Figure 2B (line 178)

The labels that were B and C are now C and D in the new figure 2, and have been organized to agree with the temporal citation of the data in the main text.

**Reviewer #2:** In the current manuscript, Fumasoni and Murray investigate how baker’s yeast can adapt to constitutive DNA replication stress, a physiological condition that is frequently observed in cancer cells. More specifically, they ask how the ploidy or the recombination competence of the yeast affect its adaptive potential and the mechanisms underlying adaptation to DNA replication stress. In order to trigger constitutive DNA replication stress, they deleted a gene (*CTF4*) that encodes a non-essential subunit of the DNA replication machinery. As expected, this genetic perturbation causes a significant decrease in reproductive fitness, providing an opportunity to explore genomic mutations that accumulate during a microbial evolution experiment and compensate the fitness loss. The Authors evolved two different types of strains: i.) a diploid yeast and ii.) a haploid yeast deficient in homologous recombination (due to the lack of *RAD52*). By characterizing the evolved strains of this current study, the Authors were able to compare the evolutionary trajectories of the above strains to that of those recombination-proficient haploids that were previously adapted by the same Authors using the very same pipeline (PMID: 32043971).

The main results are the followings:

1. At the end of the evolutionary experiment (after 1000 generations), all parallel evolved lines of all genotypes showed fitness compensation (i.e. displayed fitness increase) in the presence of DNA replication stress.

2. The extent of fitness increase showed a negative correlation with the initial fitness of the original strains, providing further evidence for the declining adaptability concept (e.g. PMID: 25815007).

3. To shed light on the mechanisms of adaptation towards DNA replication stress, whole genome sequencing was performed on the evolved lines. The Authors found several mutations, a larger fraction of them being unique to the type of strains, whereas a smaller fraction of them showed convergence at the functional modules across the strains.

4. Specifically, the evolved lines of all three types of strains (two in the current, one in the previous study) harbored genomic alterations in three biological processes, including DNA replication, DNA damage checkpoint and sister chromatid cohesion. Hence, the principles that govern adaptation to DNA replication stress are similar across all tested strains, irrespective of ploidy and recombination competence. Nevertheless, the exact genes that were mutated, and the frequency of different types of mutations largely depended on the genetic background. For instance, amplification events were less frequent in the recombination-deficient strains and recessive null mutations were rarer in diploids.

5. One-by-one reconstruction of several single mutations, affecting either of the abovementioned functional modules, increased the fitness of the ancestor strains in all cases, albeit different mutations conferred different benefits. However, the fitness benefit these mutations provided was similar across all types of strains, at least in the majority of tested mutations (3 out of 4). In addition, combination of these mutations in the ancestor strains resulted in a fitness that is highly similar to that of the evolved strains, indicating that a limited number of mutations in genome maintenance was enough to fully mirror the evolved fitness.

To sum up, the current study line-up an experimental evolution in the lab to investigate adaptation of baker’s yeast towards DNA replication stress. Nevertheless, this is something that was already done in their previous paper (PMID: 32043971), although in a different genetic background. I do understand that the main motivation of the Authors was to compare the adaptation mechanisms of genotypes with different genomic characteristics (in terms of ploidy/recombination competence), but the results are somewhat expected: different strains preferentially accumulated different types of genomic mutations, at a different frequency. As the initial stress was the same in all backgrounds, mutations in genes belonging to the same genome maintenance modules is also something that is not so surprising.

We thank reviewer #2 for their careful summary of our work and their assessment of its novelty. In response, we argue that what a reader finds surprising, or expected, is mostly subjective. For instance, based on previous literature, one could have expected diploids to adapt more slowly because most loss of function mutations are recessive and recombination-deficient strains to adapt more slowly because they cannot increase gene dosage by amplifying chromosomal regions. Instead, we see the opposite outcome, with diploids and recombination deficient strains recovering from the severe defects induced by the lack of Ctf4 at rates higher than that of haploid cells. Another example is based on the finding that in recombination-proficient strains the predominant mutation that improves sister-chromatid cohesion amplifies the genes encoding the subunits of the Scc2:Scc4 cohesin loader. It would be reasonable to argue that because this pathway is blocked in the recombination-deficient strain, that this strain would be less likely to have selected mutations that affect sister chromatid cohesion and that there should be no amplification of the cohesin loader genes. But we find that this module *is* mutated in the recombination-deficient strain and that one population has succeeded amplifying *SSC2*, albeit by a different form of recombination from the recombination-proficient haploids and diploids.

That being said, I have ambivalent feelings about the novelty of the current study from a conceptual point of view: it feels like that this work is just a follow-up study of a previous idea (PMID: 32043971, with two additional types of strains being tested) with no major conceptual advance.

We believe our work *is* a conceptual advance in the understanding of evolution in two important respects: 1) genomic features dictate which mutations are selected in response to the same genetic perturbation, but 2) the functional modules in which these mutations lie are the same despite differences in the genomic features. These advances go beyond previous work that compared the response of strains with different ploidies to the same selection in two ways: 1) we identified candidate causative mutations and verified their causality individually and in combination, and 2) we studied the consequence of blocking a particular form of mutation, alterations in gene dosage through the amplification of chromosomal segments. Reviewer 2 is entitled to argue that the finding that genomic features affect which mutations are selected is unsurprising, but there is a difference between argument and demonstration, and we find that diploid strains can still select recessive mutations (via independent mutations in two copies of a gene or homzygosis of a single recessive mutation) albeit at a lower frequency than haploid strains. In contrast, we see no way in which the finding that the same modules get mutated, despite differences in genomic features, could have been reliably predicted. We have revised the manuscript to emphasize the concepts that our evidence supports.

In addition, I also feel that the Authors have not put enough efforts into the current study that would be comparable to what they did in their previous work (PMID: 32043971). For instance, the present study do not line up any detailed biological assays to measure the effect of compensatory mutations on chromatid separation/DNA replication, etc.; something that was present in their previous work (PMID: 32043971).

We agree with the reviewer that the answers these assays provide are interesting, but in addition to the substantial effort needed to perform them in two other genomic contexts we analyzed, we believe that they lie beyond the scope of the current study. While our previous paper focused on the molecular mechanisms of adaptation, the present manuscript investigates the influence of genomic features on evolutionary dynamics and genetic paths adaptation. Under these circumstances, we find it reasonable to assume that mutations in genes whose mechanism we previously investigated in haploid (i.e the amplification of cohesin loader genes) are likely to have similar effects in other backgrounds (i.e improving sister chromatid cohesion).

In addition, I failed to find a thorough examination of the potential side-effects of the compensatory mutations in other genomic backgrounds/conditions (see major comment 4 below) that would be essential to assess their long-term prevalence in natural environmental settings.

We thank reviewer #2 for raising this interesting point. We performed key experiments of restoring Ctf4 function in strains that were evolved in its absence. The majority of these strains have fitnesses that are similar to wild type, showing that the combined effect of the compensatory mutations does not damage cells that are otherwise wild-type. This finding suggests that compensatory mutations are more likely to survive in populations even after the perturbation which triggered their selection ceases to exist, and therefore are relevant for the evolution of cell biology in nature.

Major points/questions:

1. The term genome architecture is traditionally used for the arrangement of genes, regulatory elements in the genome (PMID: 18929678) or to refer to the spatial arrangement of chromosomes within the nuclear volume (https://doi.org/10.1016/S1067-5701(98)80021-1). However, in this work (line 44-46), it is used to refer to the “ploidy, rates of recombination, and the activity of mobile genetic elements”, which is a bit odd to this Reviewer. Since this term is also used in the title, it can be misleading for the scientific community, as spatial arrangement of genes/chromosome was not tested in this work at all. I suggest replacing this term with a more appropriate one. On a side note, the Authors also mention the activity of mobile genetic elements among the features of genome architecture, but obviously, adaptation of a strain that has a modified activity of mobile genetic elements has not been performed here.

See the response to reviewer 1, in which we propose to replace genome architecture with “genomic features”. We have also removed any reference to ‘mobile genetic elements’, which were mentioned as an additional example of genome architecture in the introduction, but were not subjected to experimental analysis.

2. The extent of compensation differed across the tested strains: recombination-deficient haploid strains showed the largest fitness increase, followed by diploids and recombination-competent haploid strains. This difference can be accounted for the declining adaptability (less fit genotypes are more likely to get compensated for than those that are closer to the WT fitness). This is all OK. However, in my opinion, the Authors might misinterpret this observation. They claim that diploids evolve faster than haploids (faster appears multiple times throughout the manuscript), but we have no firm evidence for that. The extent of compensation is not the same as the speed of adaptation. I might be wrong, but to make inferences about the speed of adaptation, one would need to investigate fitness at multiple time points during the evolutionary experiment and compare the kinetics of the fitness increase across the tested strains. By doing that, one would also be able to see if there is any saturation in the fitness increase during the 1000 generations, or it is still inclining. Without doing that, we cannot objectively compare the speed of adaptation across different types of strains.

We agree that that this is an important point and we addressed it by performing the experiment suggested by Reviewer #2: We measured the fitness at multiple time points during the 1000 generations and compared the speed of adaptation across all the evolved populations (Fig 2A). Interestingly, the new data show how the differences in how much fitness increased during the entire experiment is mostly due to differences in adaptation rates over the first 100 generations (Fig 2B). In this period, diploids and recombination-deficient haploids gain substantial fitness, approaching the fitness of the evolving haploid populations. During the subsequent 900 generations, however, all the populations adapted at comparable rates. We now discuss this observation in several part of the main text:

- “*The dynamics of adaptation of strains with different genomic features was followed by measuring the fitness of samples of all populations saved during the course of the evolution experiment (Figure 2A).” (Line 167-169)*

- *“Interestingly, we found that these values mostly depend on different adaptation rates during the first 100 generations. Diploids and recombination deficient populations gain a large fraction of their final fitness during this period, approaching the initial fitness of the evolving haploid populations. Afterwards, all the populations adapted at comparable rates for the reminder of the experiment ( Figure 2B).” (Line 173-177)*

3. The Authors investigated the effect of segmental amplifications by focusing on two genes that reside on the observed segmental amplifications, namely *SCC2* and *SCC4*, members of the cohesin loader complex. By introducing an additional copy of *SCC2* into the ancestor strains, the Authors confirmed the compensatory role of this amplification, although the fitness benefit it provides seems to be a minor one compared to that of other mutations. This finding is actually rather surprising as such an increase in the gene copy number of a single subunit might have caused a stoichiometric imbalance in the complex and a subsequent fitness loss, but apparently this is not the case here. I might be wrong, but one might expect that amplifications involving both subunits of the *SCC2-SCC4* complex should make the complex more functional compared to a situation when only one is amplified. Nevertheless, it would be useful to report the number of cases (e.g. not the total, but the mean/median of all lines per backgrounds), where only a single gene or both genes were amplified in a given background during the lab evolution.

We agree with reviewer #2 that the ratio of lineages carrying amplifications of *SCC2* and *SCC4* is worth commenting on, and while we reported the total number of populations carrying such segmental amplifications, we failed to report which genes were amplified in which populations. The percentages of populations with amplifications affecting SCC2 alone, SCC4 alone, or both genes are 62.5%, 0% and 12.5% in haploids, 28.6%, 0% and 71.4% in diploids and 12.5%, 0% and 0% in recombination deficient populations. *SCC4* was never found amplified alone, but always in combination with the amplification of *SCC2*. Furthermore, the reviewer is correct in expecting a stronger increase in fitness when both SCC2 and SCC4 are amplified, something we showed in our previous work (Fumasoni and Murray, 2020, Figure 3C). We speculate that in a heterodimer, such as the cohesin loader, an initial increase in biologically active complexes could be obtained by duplicating the gene for the less abundant subunit (which we argue is Scc2). After this initial gene amplification, this subunit might be in excess, thus selecting for the subsequent amplification of the previously more abundant subunit (Scc4), which would result in a further increase in the number of functional complexes. This is consistent with the fitness we previously reported being associated with the amplification of *SCC2*, *SCC4*, or both: an extra copy of *SCC4* only increases fitness in a *ctf4Δ 2xSCC2* strain, but not in a *ctf4Δ* single mutant (Fumasoni and Murray, 2020, Figure 3C). This scenario is further corroborated by the fact that, out of the three clones isolated from the haploid population EVO2 that have amplified *SCC2*, only one also carries the amplification of *SCC4* (Table S4). A parsimonious interpretation suggests that *SCC4* was acquired later, in a background that had already amplified *SCC2*.

We have now summarized these aspects in an extra supplementary figure (Figure 3S3) and in the main text (lines 263-269):

*“SCC4 was never found amplified alone, but always in populations that had also amplified SCC2 (Figure 3-S3A). We previously showed that extra copies of SCC2 alleviate the cohesion defects associated with the absence of Ctf4 in haploid cells and increase their fitness; the additional amplification of SCC4 further increase cells’ fitness, but there is no benefit when SCC4 is amplified alone [17]. These observations are compatible with a scenario where Scc2 is the limiting subunit in the formation of active cohesin loader complexes (Figure 3-S3B).*”

4. Relating to the segmental amplifications, I have also another issue that is the involvement of several linked genes residing on the same genomic segment. Which genes were involved in these amplifications (apart from *SCC* genes)? Do we have any functional enrichment on these segments? How stable are these genomic alterations? Are there dosage sensitive genes on these segments? What could be the potential side-effects arising from the increased copy number of these linked genes? Do they affect fitness, or any other phenotypic traits, in the lack of the original DNA replication stress? Actually, all of these questions about the fitness cost of mutations can be applied to other genomic alterations, including SNPs as well. These questions could be readily investigated by introducing these mutations into the WT strain one-by-one, or alternatively, by reintroducing the deleted *CTF4* gene into the evolved lines (and hence investigating the sum effect of all compensatory mutations). On a related note, I believe it would be also important to demonstrate that the beneficial effects of the compensatory mutations are not contingent on the deletion of *CTF4*, but to DNA replication stress in general. In other words, the Authors should test the effect of compensatory mutations in the presence of DNA replication stress triggered by other genetic (deleting other important player of DNA replication) or conditional means (using drugs that induce the stress).

All of these are reasonable questions, but their thorough investigation would lie far beyond the scope of a single paper. We followed reviewer #2’s suggestions and discuss the following points:

1) To answer the reviewer’s point on the functional enrichment of the genes affected by segmental amplifications, we performed a GO-term analysis on the ones present on the chromosome segment recurrently amplified (list of genes now reported in Supplementary table 5). The analysis did not return any relevant functional enrichment, bur resulted only in ‘cellular biological process’. Similarly, we did not find any enrichment of dosage sensitive genes (DSG) in these segments (out of 115 DSG previously reported in the S. cerevisiae genome (Makanae et al., Genome Research, 2013), 14 were found on the selected segmental amplification, while 15 were predicted to be present if they were randomly distributed in segments of the same total size, p-value = 0.66 χ2 test, Supplementary table 5). We now comment on this in the main text (line 251-253):

*“We failed to detect an enrichment of any biological process GO-terms, or of reported dosage-sensitive genes on these amplified segments (Makanae et al., 2013, supplementary table S5).”*

We have instead three lines of evidence that suggest that amplifying *SCC2* and *SCC4* accounts for the increased fitness associated with the amplified segments: i) cells lacking Ctf4 have defects in sister chromatid cohesion and this is the one pair of genes on the two amplified segments that is known to cooperate in this process, ii) adding extra copies of *SCC2* and *SCC4*  to cells lacking Ctf4 improves their fitness, and iii) this fitness increase is associated with an increase in sister chromatid cohesion. We cannot exclude the existence of other genes in the amplified segments that also ameliorate the defects in *ctf4∆*, but their contribution to increasing fitness is likely to be minor, since adding an extra copy of *SCC2* and *SCC4* and making single mutations in the replication and DNA damage checkpoint modules largely recapitulates the increased fitness of the evolved populations.

2) The question of whether mutations only increase fitness in the absence of Ctf4 is an interesting one. We have followed reviewer #2 suggestion and reintegrated a wild type *CTF4* gene in a number of evolved populations. In all tested cases, reintroducing Ctf4 *increased* the fitness of the evolved clones to within 1-5% of the wild-type fitness. Altogether these results suggest that the combined effect of the mutation accumulated in response to constitutive replication stress, are either neutral or minimally detrimental in a wild-type background. Their near neutrality should increase the likelihood that these mutations will persist, even after the initial perturbation is removed, making them relevant for the diversification of cells in nature or in cancer evolution.

The following text has been added in the conclusions (line 635-641):

*“The relevance of compensatory mutations for the subsequent evolution of cells depends on their effect when the initial genetic perturbation has been removed. We found that reintegrating a wild type CTF4 gene into the evolved strains lead to fitness levels comparable to wild type cells in 8 of the 14 populations tested (Figure 6S2A). These results show that the combined effects of the mutations accumulated during the evolutionary adaptation have little effect once Ctf4 is restored, increasing the likelihood they would persist after reversal of the initial genetic perturbation.”*

3) How do other forms of replication stress interact with the mutations that were selected in the absence of *ctf4∆*: We have followed reviewer #2’s suggestion and measured the fitness of evolved populations in which Ctf4 had been restored in the presence of hydroxyurea, a drug inducing replication stress by depleting nucleotide pools. Interestingly, we have found a diverse response, with populations being less (9/14), equally (1/14), and more (2/14) fit than wild type cells (Figure 6S2B). This shows how the evolutionary response that we have characterized contain a mix of mutations which, in certain combinations can confer protection to other forms of DNA replication stress, but in others is neutral or detrimental. This is not unexpected, since different forms of DNA replication stress (nucleotides depletion, under-replication, re-replication, replication-transcription conflicts, Hills and Diffley, 2014) have different mechanisms of cytotoxicity. To improve the clarity of our conclusions, we now refer throughout the manuscript to *ctf4Δ*-induced DNA replication stress as “constitutive DNA replication stress” or to “a form of DNA replication stress”, to distinguish it from other forms of stress.

The following text has been added in the conclusions (line 641-647):

*“When the same populations were tested in the presence of Hydroxyurea, a drug which depletes nucleotide pools by inhibiting the ribonucleotide reductase, we found a diverse response, with populations to which Ctf4 had been restored being less (9/14), equally (1/14), and more (2/14) fit than wild type cells (Figure 6S2B). The adaptation to ctf4Δ-induced constitutive DNA replication stress can therefore be effective against other type of stresses perturbing DNA replication, but this is likely contingent on the particular mutations accumulated by the strains, and the nature of the replication defects applied to them.”*

5. The Author’s main focus is on the recurrently mutated genes that accumulated in all three types of strains. They also demonstrated that these mutations, representing only a small fraction of all mutations, are largely responsible for the fitness compensation. For this reason, they argue that the large fraction of the mutations, those that are strains-specific, play only a marginal role in adaptation. On a side note, when they reconstructed such mutations (affecting one of the three genome maintenance modules) that were never seen in a given background, they happened to see beneficial fitness effect. Thus, I would not exclude the possibility that emergence of unique mutations only on one genomic background (including those that are not involved in the genome maintenance) would not be relevant from an evolutionary point of view. In my opinion, it would be informative to expand the text accordingly by adding a short overview of the type of genes that were uniquely mutated in each specific type of strains. This is something that is much expected when the Authors would like to pursue the differences of adaptation between different genetic backgrounds.

We did not find any enrichment in GO-terms associated with the genes significantly selected uniquely in strains with a specific genomic feature (haploid, diploids or recombination deficient). Nevertheless, we agree with reviewer #2 that among these genes, some mutations are likely contributing to evolutionary adaptation in a particular strain. We explicit mention this possibility in the main text, using mutations in *FOB1* as an example (line 523-530):

*“**Nevertheless, we cannot exclude the possibility that mutations that are only found in the presence of a particular genomic feature may play important roles in adaptation. One example is mutations in the gene encoding the replication fork barrier Fob1, which coordinates the replication of the tandem repeats present at the rDNA locus. In the absence of Ctf4, Fob1 can induce replication fork collapse, resulting in double strand breaks which can be rescued by recombination mediated processes. Inactivating Fob1 is thus specifically beneficial in recombination-deficient cells which would otherwise accumulate irreparable chromosome breaks (Sasaki and Kobayashi, 2017).”*

Minor points/questions

1. Is there any overlap of the mutated genes and those that are in suppressor interaction with *CTF4*?

Interestingly, we found no significant overlap. None of the 50 genes considered significantly selected in this study appears in the stringent list (27 genes, score > 0.16, p-value < 0.05) of positive genetic interactions on the cellmap.org central repository. Only three genes (*BST1*, *WHI2* and *KRE29*), none of which are major contributors to the evolved fitness according to our study, show an overlap when an intermediate cutoff (324 genes, score > 0.08, p-value < 0.05) is applied to the list of genes with positive interactions. This is very close to the level of overlap predicted by chance (50*324/6000 = 2.7). We have now included the following sentence in the main text (Line 230-233):

“*Interestingly, we found no significant overlap (p=0.48, hypergeometric test) between these genes and those previously reported to have positive genetic interactions with ctf4Δ mutants (Usaj et al.,2017), highlighting how systematic analysis of genetic interactions fails to predict the outcome of an evolutionary repair process.”*

2. The Authors mention that diploidization is frequently observed during experimental evolution. Have the Authors investigated this possibility in the case of their haploid strains (both in the current and the previous study)?

We measured DNA content as a proxy of ploidy in all the evolved populations and found no change by the end of the experiment (except for *EVO14*, where we reported that a diploid population became haploid, likely due to a contamination event). We provide the data in a figure for reviewers only.

3. In order to measure fitness, The Authors used competition experiment. For this purpose, they introduced a fluorescent reporter into the promoter region of the *ACT1* gene (a gene being both haploinsufficient and essential) of the strains. Have the Authors tested if insertion into this genomic locus does not perturb actin function and does not have any side-effects?

The insertion of the plasmid carrying the fluorescent reported is designed to generate a duplication of the *ACT1* promoter, with one copy driving the expression of the native *ACT1* gene, and the other copy independently expressing the fluorophore. We have no reason to believe that actin would be perturbed in such system but have done no biochemistry or cell biology to test this possibility. To prevent any possibility of a bias in the fitness measurement induced by the fluorophore, all ancestor strains employed in the experimental evolution were equipped with a similar system (expressing mCherry instead of yCerulean). The fitness difference between the ancestor cells against the respective reference is often very low (e.g 0.03% ±0.06 of wild type expressing mCherry against wt expressing yCerulean), is always reported in the source data files, and subtracted from the sample measurements to account for any potential fitness difference induced by the fluorophore expression system.

**Have all data underlying the figures and results presented in the manuscript been provided?**
Large-scale datasets should be made available via a public repository as described in the *PLOS Genetics* [data availability policy](https://urldefense.proofpoint.com/v2/url?u=http-3A__journals.plos.org_plosgenetics_s_data-2Davailability&d=DwMGaQ&c=WO-RGvefibhHBZq3fL85hQ&r=TldrIYF6lskrQP6U5T_8DH9C6gUJXGUi0Gga6NNiF24&m=yXTh65OlSBOZdTSOt2MHmjd-4h55zHfpicll8mil8os&s=DPE4Iu3MnYdtxjBAXF3_xhcn1Y9mBCk_gjBBERF5pD0&e=), and numerical data that underlies graphs or summary statistics should be provided in spreadsheet form as supporting information.

Reviewer #1: Yes

Reviewer #2: Yes

PLOS authors have the option to publish the peer review history of their article ([what does this mean?](https://urldefense.proofpoint.com/v2/url?u=https-3A__journals.plos.org_plosgenetics_s_editorial-2Dand-2Dpeer-2Dreview-2Dprocess-23loc-2Dpeer-2Dreview-2Dhistory&d=DwMGaQ&c=WO-RGvefibhHBZq3fL85hQ&r=TldrIYF6lskrQP6U5T_8DH9C6gUJXGUi0Gga6NNiF24&m=yXTh65OlSBOZdTSOt2MHmjd-4h55zHfpicll8mil8os&s=T7MY2hTKTF8YooAePDnLQkULGkLSeIegpQMgoolVTjo&e=)). If published, this will include your full peer review and any attached files.

If you choose “no”, your identity will remain anonymous but your review may still be made public.


**Do you want your identity to be public for this peer review?** For information about this choice, including consent withdrawal, please see our [Privacy Policy](https://urldefense.proofpoint.com/v2/url?u=https-3A__www.plos.org_privacy-2Dpolicy&d=DwMGaQ&c=WO-RGvefibhHBZq3fL85hQ&r=TldrIYF6lskrQP6U5T_8DH9C6gUJXGUi0Gga6NNiF24&m=yXTh65OlSBOZdTSOt2MHmjd-4h55zHfpicll8mil8os&s=ntJFXkZrHGBxkoEoivaSdP8KxtXwOwmudVpQhBqfnKs&e=).

Reviewer #1: No

Reviewer #2: No

*In compliance with data protection regulations, you may request that we remove your personal registration details at any time.* [*(Remove my information/details)*](https://urldefense.proofpoint.com/v2/url?u=https-3A__www.editorialmanager.com_pgenetics_login.asp-3Fa-3Dr&d=DwMGaQ&c=WO-RGvefibhHBZq3fL85hQ&r=TldrIYF6lskrQP6U5T_8DH9C6gUJXGUi0Gga6NNiF24&m=yXTh65OlSBOZdTSOt2MHmjd-4h55zHfpicll8mil8os&s=sFHz6jFTTBjk2qMSdM1LLcZSj2jtT8WLQSBNBYuFrog&e=)*. Please contact the publication office if you have any questions.*
